# Combination of Hypomorphic Mutations of the *Drosophila* Homologues of *Aryl Hydrocarbon Receptor* and *Nucleosome Assembly Protein* Family Genes Disrupts Morphogenesis, Memory and Detoxification

**DOI:** 10.1371/journal.pone.0094975

**Published:** 2014-04-15

**Authors:** Boris A. Kuzin, Ekaterina A. Nikitina, Roman O. Cherezov, Julia E. Vorontsova, Mikhail S. Slezinger, Olga G. Zatsepina, Olga B. Simonova, Grigori N. Enikolopov, Elena V. Savvateeva-Popova

**Affiliations:** 1 Kol'tsov Institute of Developmental Biology, Russian Academy of Sciences, Moscow, Russia; 2 NBIC, Moscow Institute of Physics and Technology, Moscow, Russia; 3 Pavlov Institute of Physiology, Russian Academy of Sciences, St. Petersburg, Russia; 4 Engelhardt Institute of Molecular Biology, Russian Academy of Sciences, Moscow, Russia; 5 Cold Spring Harbor Laboratory, Cold Spring Harbor, New York, United States of America; Alexander Fleming Biomedical Sciences Research Center, Greece

## Abstract

Aryl hydrocarbon receptor is essential for biological responses to endogenous and exogenous toxins in mammals. Its *Drosophila* homolog *spineless* plays an important role in fly morphogenesis. We have previously shown that during morphogenesis *spineless* genetically interacts with *CG5017* gene, which encodes a nucleosome assembly factor and may affect cognitive function of the fly. We now demonstrate synergistic interactions of *spineless* and *CG5017* in pathways controlling oxidative stress response and long-term memory formation in *Drosophila melanogaster*. Oxidative stress was induced by low doses of X-ray irradiation of flies carrying hypomorphic mutation of *spineless*, mutation of *CG5017*, and their combination. To determine the sensitivity of these mutants to pharmacological modifiers of the irradiation effect, we irradiated flies growing on standard medium supplemented by radiosensitizer furazidin and radioprotector serotonin. The effects of irradiation were investigated by analyzing leg and antenna morphological structures and by using real-time PCR to measure mRNA expression levels for *spineless*, *Cyp6g1* and *Gst-theta* genes. We also examined long-term memory in these mutants using conditioned courtship suppression paradigm. Our results show that the interaction of *spineless* and *CG5017* is important for regulation of morphogenesis, long-term memory formation, and detoxification during oxidative stress. Since *spineless* and *CG5017* are evolutionary conserved, these results must be considered when evaluating the risk of combining similar mutations in other organisms, including humans.

## Introduction

Aryl hydrocarbon (dioxin) receptor (Ahr), by triggering a metabolic detoxification response in vertebrates, acts as a key factor of the cellular response to endogenous and exogenous toxins and may play a larger role in the generalized cellular stress response. Ahr is a transcription factor containing a structural bHLH-PAS DNA-binding domain cytoplasmic protein. It is normally present in the cytoplasm; however, when it binds endogenous or exogenous toxins (e.g., dioxins), it is activated and translocated into the nucleus, where it binds another bHLH-PAS-protein, the Aryl hydrocarbon receptor nuclear translocator (Arnt). The Ahr::Arnt heterodimer binds to a specific motif (Xenobiotic Response Element; XRE) in the promoters of its target genes and controls their transcription [1]. Ahr has other functions besides being involved in detoxification and stress response, and null and hypomorphic mutations of *Ahr* disrupt embryonic development and affect physiological functions in mammals [2]–[5].

*Ahr* gene is highly conserved between vertebrates and invertebrates. Unlike their vertebrate homologs, invertebrate Ahr do not show the ability to bind toxic agonists (e.g., a reference dioxin cogener 2,3,7,8-tetrachlorodibenzo-p-dioxin; TCDD) [6], [7]. However, they carry important developmental functions. For instance, mutations in the *Drosophila melanogaster* homologue of mammalian *Ahr*, *spineless* (*ss*) [8], affect leg and eye development of the fly and disrupt development of neuronal dendritic structure. In *Caenorhabditis elegans* mutations in the *Ahr* homologue *Ahr-1* results in abnormal neuronal differentiation [7], [9]. Appearance of a new function of the Ahr proteins in vertebrates, - their ability to bind toxic agonists, may reflect evolutionary directed gains of serving as adaptors in response to environmental pollutants [10].

We have previously generated a novel *D. melanogaster ss* mutant line by crossing flies carrying a hypomorphic *ss^a40a^* mutation with flies from the supermutagenic dysgenic *w oc/FM4* strain. The resulting *ss^aSc^* flies, in addition to the mutation in the *ss* locus, carry a *P-element* insertion in the region of the *CG5017* locus [11], [12]. *ss^aSc^* flies differ from the original *ss^a40a^* flies by an augmented manifestation of the mutant *ss* phenotype and decreased levels of expression of the *CG5017* gene. The product of the *CG5017* gene is homologous to proteins of the Nucleosome assembly protein (NAP) super-family, which are important for the histone transport to the cell nucleus and nucleosome assembly [13]. These proteins belong to the chaperone family, whose members are associated with nucleosomes [14]. The open reading frame of the fly *CG5017* encodes a 283 amino acid long protein, most of which (amino acids 46 to 252) represents a defined domain. The null mutation of *CG5017* disturbs spermatid elongation [15]. Two hypomorphic mutations of the *CG5017* gene have been isolated; both of them do not lead to morphological defects, but affect memory formation in flies [16], [17]. Since the *Ahr* genes involved in morphogenesis and stress response and the *CG5017* gene involved in the establishment of long-term memory, we sought to investigate interactions between these genes in each of these pathways in *Drosophila*.

Since the *Ahr* gene, besides its role in the regulation of morphogenesis, also plays a role in cellular reaction to toxin exposure, and, in particular, to ionizing radiation [18], [19] we studied the response to the effects of low doses of ionizing radiation and the capacity for memory formation in flies carrying a combination of hypomorphic mutations in the *Ahr* and *CG5017* genes. Our results show new genetic interactions between the *Ahr* and *CG5017* genes in both morphogenesis and behavior. While this study concerns a model organism, our results imply that these synergistic interactions should be taken into account when considering the risk of radiation and oxidative stress exposure in humans.

## Materials and Methods

### Fly Stocks and Rearing Conditions

We used wild type *Canton S* and mutant *ss^a40a^* obtained from the Bloomington *Drosophila* stock center, s*s^aSc^* [11], *milkah-1* [16], and *ss^a40ahm^* strains of *Drosophila melanogaster*. *ss^a40ahm^* strain, used in this study, originated from the *ss^a40a^* line as a result of a spontaneous mutation, and has less pronounced phenotype than *ss^a40a^*. Crossover combination *ss^a40ahm^ milkah-1* was generated by crossing *ss^a40ahm^* and *milkah-1* flies. In genetic experiments and experiments studying the effect of ionizing radiation on the fly development we used Formula 4-24 medium (Carolina Biological Supply, Burlington, NC, USA) supplemented with yeast paste. When required, furazidin and 5-hydroxytryptamine (Sigma-Aldrich, Saint Louis, MO, USA) were added to the medium at 80 ml/L concentration. Larvae and flies were kept at room temperature (23°C). During behavioral experiments flies were reared on standard medium (cornmeal/sugar/yeast) at a temperature of +25±0,5°C and 45%–60% humidity with a 12-h light/dark cycle.

### Irradiation of *Drosophila melanogaster* Larvae and Adult Males

A RUM-17 X-ray irradiator was used as the source of ionizing radiation, with the radiation dose depending on the time of exposure and the distance between the irradiator and the irradiated object. To evaluate the effect of radiation on the transcription levels of the *Cyp6g1*, *ss*, and *CG1681* (*GST-theta*) genes in *D. melanogaster* males we used two different irradiation regimens. In the first regimen the flies were irradiated for 15 seconds, at a distance of 47 cm. In the second regimen flies were irradiated for 60 seconds at a distance of 74 cm.

### Evaluation of the Toxicity of Ionizing Radiation on the Development of Distal Limb Structures of *Drosophila melanogaster*

Larvae were irradiated during the second half of the third instar stage. Following metamorphosis, all imagoes were collected, fixed in ethanol and the effect on the limb structures was evaluated by analyzing the number of the leg tarsal segments and the degree of transformation of the arista into the tarsus (Table S1).

### Measurement of the mRNA Expression Levels of *Cyp6g1, ss* and *CG1681* Genes Following Exposure of *D. melanogaster* Imagoes to Ionizing Radiation

The effect of ionizing radiation on the transcription *Cyp6g1*, *ss*, and *CG1681* (*GST-theta*) genes was evaluated at the imago stage. Around 60 adult males were irradiated two days after emerging. 6–7 hrs after irradiation flies were frozen in liquid nitrogen and total RNA was extracted using Trizol reagent (Sigma-Aldrich, USA) according to the manufacturer's specifications. RNA Samples were treated with DNase (DNA-free kit, Applied BioSystems, Life Technologies, USA) according to the manufacturer's protocol to remove genomic DNA contamination. cDNA was synthesized from 1–5 µg of total RNA, using a cDNA synthesis kit with oligo-dT priming (Thermo Fisher Scientific, USA). The levels of mRNA expression of the *Cyp6g1*, *ss*, and *CG1681* genes were measured with real-time PCR using TaqMan probes (Syntol, Russia). All reactions were carried out in triplicate. Real-time PCR was conducted using an ABI Prism 7500 Sequence Detection System (Applied BioSystems, Life Technologies, USA). Differences in expression were determined using the comparative Ct method described in the ABI user manual. *Rpl32* was used as the endogenous control for normalization. The following primer pairs and probes were used for amplification: for the *Rpl32* gene primers Rpl32dir 5′-CCAGCATACAGGCCCAAGATC-3′, Rpl32rev 5′-ACGCACTCTGTTGTCGATACC-3′, probe – FAM- CGCACCAAGCACTTCATCCGCCAC-BHQ1; for the *Cyp6g1* gene primers Cyp6g1f 5′-GCGATCCATTGGGCTATAAT-3′, Cyp6g1r 5′-CCAATCTCCTGCATAAGGGT-3′, probe - FAM- TCGCACCAAGCTGACTCCCG-BHQ1; for the *CG1681* (*GST*-*theta*) gene primers CG1681f 5′-TTCGCACCCACTCTAGTCAC-3′, CG1681r 5′-GCTCGATTGGTTCAGGAAAT-3′, probe - FAM- TCAACGAGATGTCGCAGCCACTC-BHQ1, for the *ss* gene primers SSdir 5′- CGAAGGCGACGCAACGG-3′, SSrev 5′- GATGCCGCTTTGATGGATTGC-3′, probe – FAM-AGCCTGAAGCCGCCGCCCAAG-BHQ1.

### Evaluation of Learning and Memory Formation in *D. melanogaster* Males

To evaluate long-term memory formation in *Drosophila* males we used conditioned courtship suppression paradigm (CCSP) [21]. In *Drosophila* the courtship ritual begins as the male, attracted by the female pheromone, initiates successive stages of courtship (orientation and pursuit, wing vibration, i.e. courtship song, licking, attempt to copulate), each of which leads to increased receptivity of the female and slowing of its movements to ensure copulation. A fertilized female will avoid new copulations during the subsequent 10–12 day period of egg laying: upon male's attempt it will extrude her ovipositor and release aversive pheromone which act as an antiaphrodisiac. The male has therefore to learn to recognize the type of female that it encounters. On meeting a fertilized female, the male, based on his former individual experience, continues or interrupts its courtship ritual. Therefore, a 30-minute courtship of fertilized female by a naïve male (defined here as training) suppresses male's subsequent courtship of both virgin and fertilized females and allows for testing short-term memory. In the first case it lasts for 1 hr, in the second case – for 8 hrs. A more prolonged, 5-hrs training, allows for testing long-term memory. In this case the tests can be performed immediately, 1, 2 and 8 days following the 5-hrs training. Either at training or testing, the courtship index (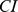) is calculated as the percentage of time spent in courtship. Strain-dependent variations in the 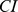 do not lead to a change in the structure of courtship behavior. However, a Learning Index (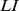) is calculated to overcome these strain-specific variations of 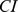
s and allow comparisons between different strains.

Following eclosion, flies were sorted according to sex without subjecting them to anesthesia. Males of the lines under investigation were placed individually on yeast-raisin medium. As courtship subjects for all lines analyzed we used *Canton S* females fertilized one day prior to the experiment at the age of five days. Studies were performed using adult five-day old flies during the first half of the day. Learning and testing were performed in Plexiglas experimental chambers (diameter – 15 mm, height – 5 mm).

A five-day old male with no experience of sexual contact, was placed in the experimental chamber along with a fertilized five-day old *Canton S* female. Memory tests were carried out at various times. As a control, males lacking any experience of sexual contact were used. An ethogram of the male's behavior was recorded over a period of 300 seconds. The following courtship elements (orientation and pursuit, vibration, licking, attempted copulation), as well as noncourtship behaviors (locomotion, preening, rest) were recorded. Recording started 45 seconds after placing the fly in the chamber. For each group (control, immediately following training and at given time intervals after training) 20 flies were tested. Registration and analysis of the results was carried out using a specially developed computer program (developed by N.G. Kamyshev).

For each male, the courtship index (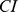), i.e. the time of courtship of the female by the male, expressed as a percentage of total observation time, was calculated. For quantitative evaluation of the results of learning, the following formula was used to calculate the learning index (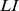):
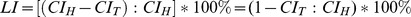
where 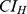 and 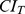
are the average 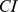 for independent cohorts of males with no experience of sexual contact, and of trained males [21]–[23].

Long-term memory was analyzed in conditioned courtship suppression paradigm [24] with some modifications. A five-day old male of the strain to be studied, with no experience of sexual contact, was placed together with a fertilized five-day old *Canton S* female into a beaker containing medium (volume of free space – about 3 cm^3^) and left for 5 hours. Memory was tested at different time intervals: immediately after training and 1 day after training, using new fertilized, 5-day old *Canton S* females.

### Statistical Analysis

Detailed comparisons of statistical procedures ever used for analysis of data obtained in CCSP (approaches based on normal theory, Wilcoxon's tests and randomization analysis [22]) are described in [21]. Briefly, randomization analysis is based on looking through all possible hypothetical experimental plans and calculating the probability of getting the value of statistic of interest obtained in the real experiment by chance [22]. A set of hypothetical experimental plans is generated by permutations between samples. In case of two samples, for example, the algorithm is as follows. Two data sets, representing each sample, are joined into one data vector. Then, the latter is used to form two data sets (with the same size as real samples) by chance. In practical terms, a random number generator is used to define which elements from the joint data vector will belong to the first data set, the others belonging by default to the second. This procedure results in creating one random experimental plan. If it is conceivable to create all possible experimental plans, where each plan is unique, such a test is called an exact randomization test. When the number of possible experimental plans is too large, it is sufficient to generate a sample of random experimental plans. The sampled randomization test with the number of random experimental plans generated (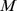) equal to 10,000 appeared to be more appropriate to the analysis of learning/memory data [21]. For each of the random plans, the statistics of interest (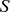) was calculated and compared with that obtained in the real experiment (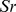). The greater the number of cases for which 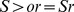, the more probable it is that the result of a real experiment may be obtained by chance under the existing data variability (that is, the greater the probability of the null hypothesis). The number of hypothetical experiments (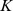) promoting 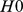, that is, fitting the condition 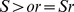
(for the one-sided test) or 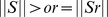 (for the two-sided test), was counted. The minimum significance level, 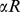, at which the null hypothesis might be rejected, was determined as 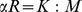. The null hypothesis was rejected if 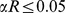 [21].

Therefore, statistical comparisons of behavioral data were made by a randomization test [21]–[22], by direct computing the probability of rejection of the null-hypothesis 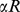. The sampled randomization test with 10,000 permutations was used. The null-hypothesis was rejected at 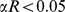. Only pair-wise comparisons were made: performance of each mutant strain was compared to that of the wild type *Canton S* strain. The two-sided test was used to compare courtship indices and various element indices between the experimental variants. The two-sided test was used to compare courtship indices between the experimental variants. The program Drosophila Courtship Lite program (Nikolai Kamyshev, 2006) was used to perform randomization tests. The program is freely available from the author (nkamster@gmail.com) upon request.

The statistical significance of the differences between samples for Real-time PCR experiments were evaluated using the REST program software (Qiagen, USA) [20] using the pair wise fixed reallocation randomization test with 2,000 permutations. A P-value of less than 0.05 was considered significant.

## Results

### *ss* and *CG5017* Genes Interact During Fly Morphogenesis

To confirm the interaction between the *Drosophila ss* and *CG5017* genes (both of which are mutated in the *ss^aSc^* line), we combined two independently derived hypomorphic mutations at the *ss* and *CG5017* loci in an independent cross. We used a hypomorphic *ss^a40ahm^* line which was independently derived from the original mutant *ss^a40a^* flies and is characterized by a less pronounced transformation of the distal segments of the antennae to the tarsus. We also used an independently derived *milkah-1* line carrying a mutation in the *CG5017* locus. This mutant does not display obvious morphological defects (in particular, in the leg and antennal structures), but is characterized by disruption of long-term memory [16].

**Figure 1:**
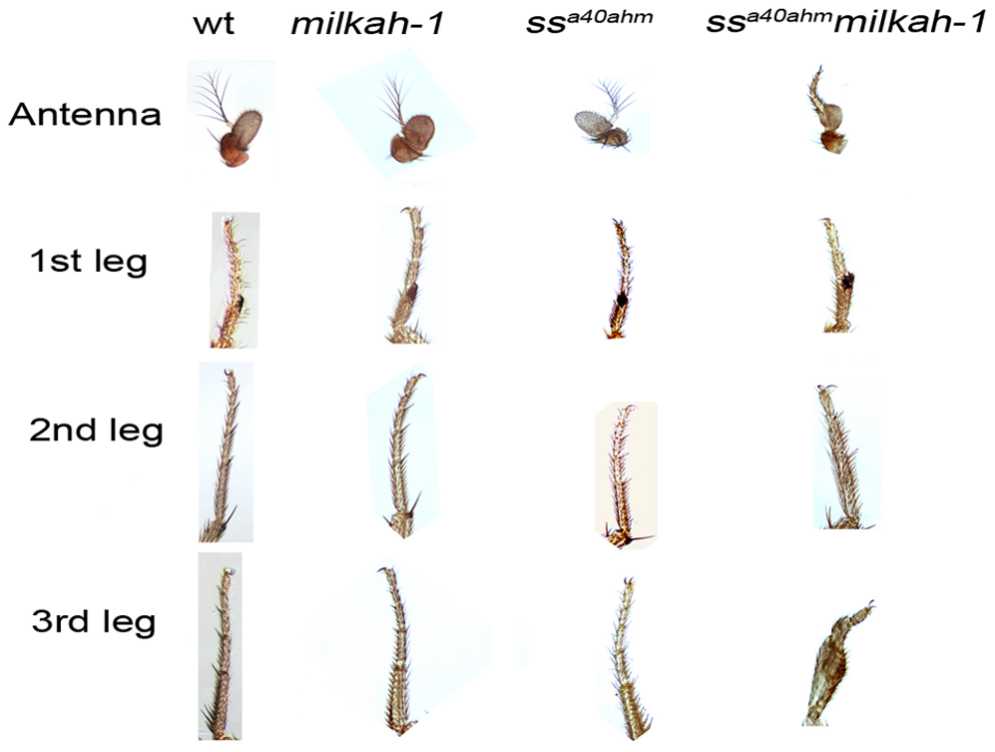
Wild type and mutant leg and antenna phenotypes. Micrographs show the normal morphology of the tarsal structures of the wild type *Canton S*, *ss^a40ahm^* and *milkah-1* flies. Antennal structures of the *milkah-1* mutant flies do not show any difference from the wild type. The distal segment of the antenna – the arista - of the *ss^a40ahm^* flies presents a certain thickening of the proximal end. The arista of the *ss^a40ahm^milkah-1* hybrid flies suffered homeotic transformation into an unsegmented tarsus. The tarsal structures of the *ss^a40ahm^milkah-1* hybrids show altered segment fusion.

Resulting *ss^a40ahm^milkah-1* hybrid flies carried mutations in both loci (confirmed genetically and by PCR) and differed from the initial *ss^a40ahm^* flies by a sharp increase in the *ss* phenotype (Fig. 1), with changes observed not only in the antennal structures but also in the tarsal segments of the legs.

Overall, the manifestation of the *ss* phenotype in the *ss^aSc^* and *ss^a40ahm^milkah-1* flies was similar, confirming the interaction of the *ss* and *CG5017* genes in the regulation of limb morphology in *Drosophila*.

### Interaction between *ss* and *CG5017* Genes Potentiates Response to Radiation

Interaction of the *ss* and C*G5017* genes in development raises a question of their potential interactions in other contexts where the function of these genes is important, in particular, for the regulation of the metabolic response to toxins, radiation and cellular stress, and for memory formation. We addressed the potential role of these genes in response to radiation by examining the impact of low doses of ionizing radiation on morphogenesis in flies carrying hypomorphic mutations in the *spineless* and *CG5017* loci. We compared the leg and antennal structures in wild type (*Canton S*), *ss^a40a^*, *milkah-1*, and *ss^aSc^* flies after exposing third instar larvae to low (1–10 R) and high (500 R) doses of radiation.

**Figure 2:**
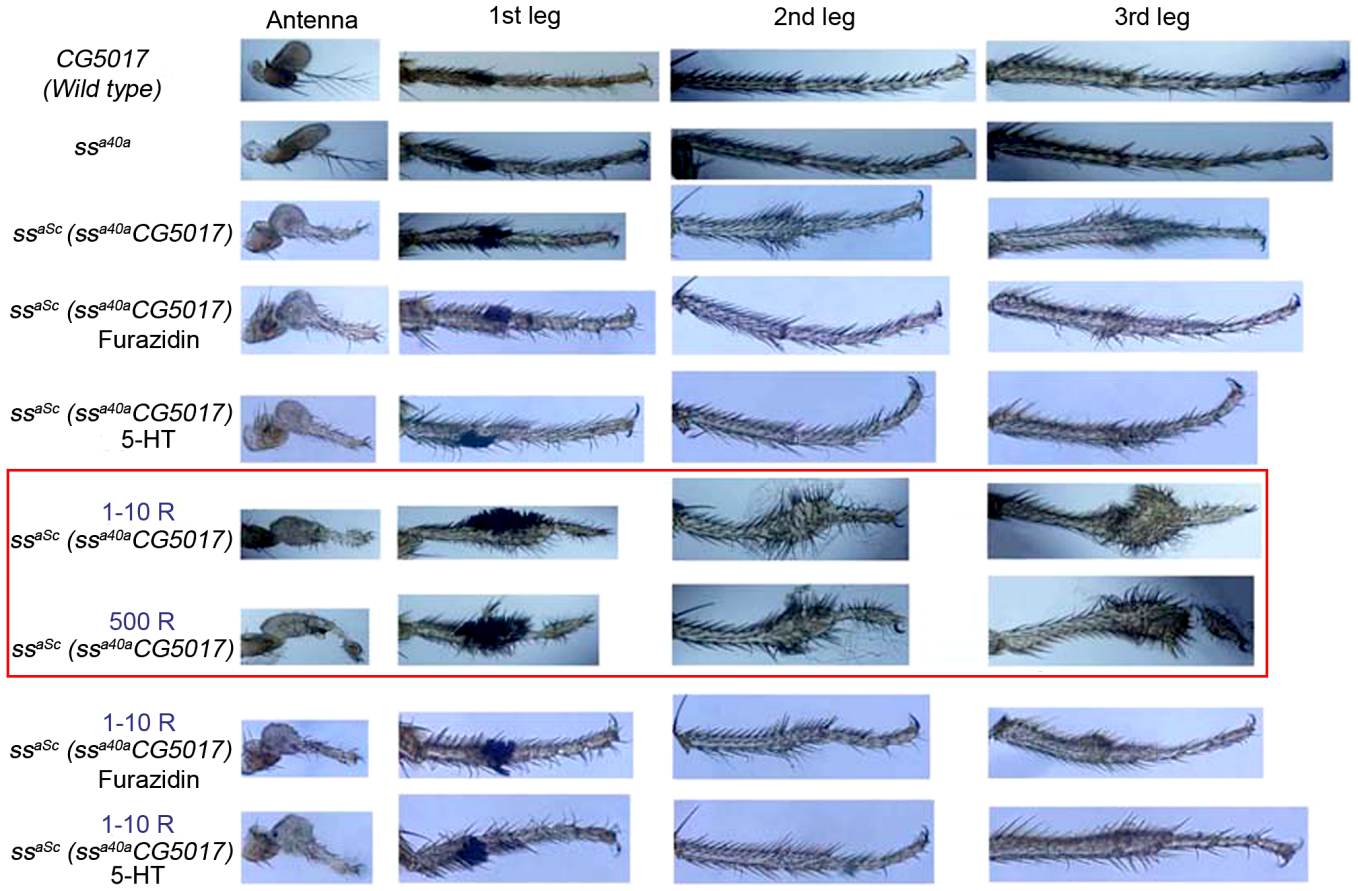
Wild type and mutant antenna and leg in flies in normal conditions and following irradiation. Simultaneous combination of mutations at both *ss* and *CG5017* loci increases sensitivity to even low doses of X-ray radiation (1 and 10 R), which is manifested as an increase in the mutant phenotype (photographs within the red frame).

Analysis of the leg and antenna structures of imagoes of *Canton S* and *milkah-1* and *ss^a40a^* mutants irradiated during the third instar phase did not reveal any obvious effect of X-ray radiation on the morphogenesis of their limbs (Fig. 2, top three rows; note that under normal conditions the leg and antenna structures of the *milkah-1* mutants do not differ from those of the wild type flies). However, irradiation of larvae whose genomes carry hypomorphic mutations in the *ss^a40a^* and *CG5017* genes, leads to a disruption of development even at the low doses – 1–10 R (Fig. 2). These results suggest that combination of the *ss* and *CG5017* genes greatly potentiates the developmental morphogenetic response to low doses of radiation. Certain chemical compounds can modify cellular and organismal response to radiation. We therefore asked whether such compounds will alter the response of mutant flies to low doses of X-ray radiation. We examined the effect of a nitrofurane derivative furazidin and of 5-hydroxytryptamine (serotonin). Nitrofurane derivatives belong to the group of the radiosensitizing agents [25], whereas 5-hydroxytryptamine acts as a radioprotector [26]. Neither furazidin, nor 5-hydroxytryptamine, when added to the media, affected limb morphogenesis of *Canton S*, *milkah-1* and *ss^a40a^* flies, with or without irradiation (Fig. 2 and data not shown). However, addition of 5-hydroxytryptamine into the medium of larvae carrying a combination of hypomorphic mutations in the *ss* and *CG5017* genes improved leg segmentation (Fig. 2). The action of 5-hydroxytryptamine was particularly pronounced when combined with irradiation (Fig. 2, Table S1). Addition of furazidin to the larval medium also resulted in improved leg segmentation in flies carrying a combination of hypomorphic mutations in the *ss* and *CG5017* genes, with or without radiation (however, it resulted in the death of 70% of irradiated flies) (Fig. 2, Table S1). Thus, compounds that can modify the cellular response to radiation are also capable of modifying morphogenetic changes in hybrid mutant flies exposed to irradiation.

### *Cyp, GST*, and *ss* Genes Are Induced in Response to Radiation

Increase in superoxide levels is the major cellular metabolic response to exposure to ionizing radiation; this in turn leads to generation of secondary and tertiary noxious derivative compounds [27]. These deleterious events are countered by increased activity of genes involved in detoxification metabolic response, such as cytochromes 450 (CYPs) and glutathione S-transferases (GSTs). We therefore examined the radiation response of *Cyp6g1* (a member of the Cyp gene family) and GST-theta/CG1681 (a member of the Gst gene family) genes, which are known to be activated in response to various toxins and irradiation [28], [29].

The regulatory elements of these genes contain functional 8-nucleotide XRE motifs, suggesting that their expression may be dependent on the *ss* gene expression. The *ss* gene also contains XRE motifs, suggesting the possibility of autoregulation.

**Figure 3:**
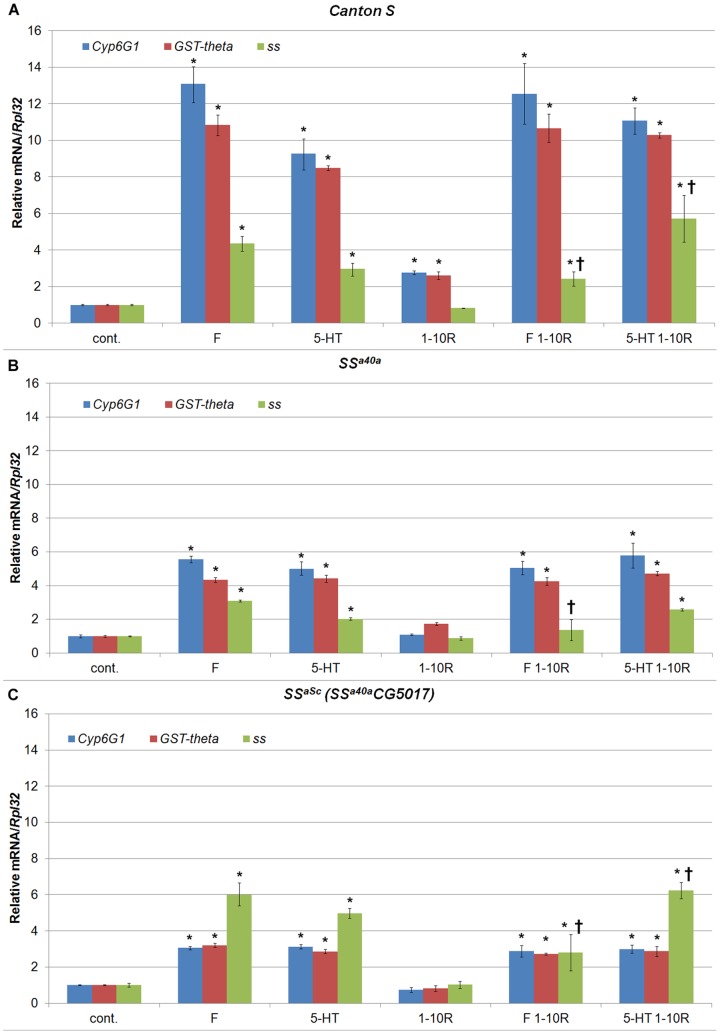
Effect of X-rays and pharmacological agents on Cyp6g1, GST-theta (CG1681) and ss mRNA expression. The relative level of expression of mRNA was measured using real-time PCR. A, B, C – level of mRNA expressed in *Canton S*, *ss^a40a^* and *ss^aSc^* flies respectively. As a control, we used RNA from flies, which have not been exposed to radiation (cont.). F, 5-HT – level of mRNA expressed in flies, grown on medium with added furazidin (F) and 5-hydroxytryptamine (5-HT), respectively; R – radiation dose in Roentgens; 1-10R, level of expressed mRNA of the genes under study in flies irradiated with a dose of 1-10 R. F1-10R, 5-HT1-10R – level of expressed mRNA of the genes under study in flies grown on medium with added F or 5-HT and radiated with 1-10 R, respectively. The bars show the level of mRNA expression. The error bars represent the standard error of the mean of triplicate experiments. * - P<0.05, compared to control group, 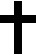 - P<0.05, compared to F or 5-HT group.

We irradiated adult *Canton S*, *ss^a40a^*, *milkah-1* and *ss^aSc^* males raised on standard medium or on medium containing furazidin or 5-hydroxytryptamine, and used PCR to determine the levels of expression of the *Cyp6g1* and *CG1681* genes, as well as of the *ss* gene (Fig. 3).

Flies of all four lines responded to irradiation, furazidin, 5-hydroxytryptamine, or their combination with increased expression of the *Cyp6g1*, *CG1681*, or *ss* genes (Fig. 2; response of the *milkah-1* flies was virtually identical to that of the wild type). The strongest response was observed in wild type *Canton S* flies, where the levels of *Cyp6g1 and CG1681* transcripts increased over ten-fold upon addition of furazidin to the medium, 8–9 fold upon addition of 5-hydroxytryptamine and, to a lesser degree, in response to X-ray irradiation. While in *ss^a40a^* and *ss^aSc^* flies the levels of *Cyp6g1* and *CG1681* transcripts also increased in response to the addition of furazidin and 5-hydroxytryptamine, the increase was several times less than in the wild type or *milkah-1* flies. Furthermore, mutation in the *ss* gene abrogated the *Cyp6g1* and *CG1681* response to radiation. Similar changes were observed when radiation was combined with furazidin and 5-hydroxytryptamine. *ss* gene itself was strongly activated by furazidin and 5-hydroxytryptamine, with or without radiation, but not by radiation itself (although combination of radiation with furazidin or 5-hydroxytryptamine changed its level of expression). Mutation in the *ss* gene did not have clear effect on its transcription. Together, these results suggest that xenobiotics (note that concentration of 5-hydroxytryptamine delivered to flies was much larger than its endogenous levels during development or in the adult) and radiation induce expression of detoxification-related genes *Cyp6g1* and CG1681 and that *ss* gene is necessary for this induction.

### *ss* and *CG5017* Genes Are Involved in Learning and Memory

Since the *CG5017* gene mutation was initially detected as one disrupting learning and memory in flies [16], [17], we evaluated its interaction with the *ss* gene mutations not only in developmental regulation and response to toxin exposure, but also in memory formation. The involvement of the tryptophan pathway in the regulation of *Ahr* gene expression and the mechanisms of memory formation [18], [30], [31] provides additional support for a possible behavioral effect of the *ss*-*CG5017* interaction.

**Figure 4:**
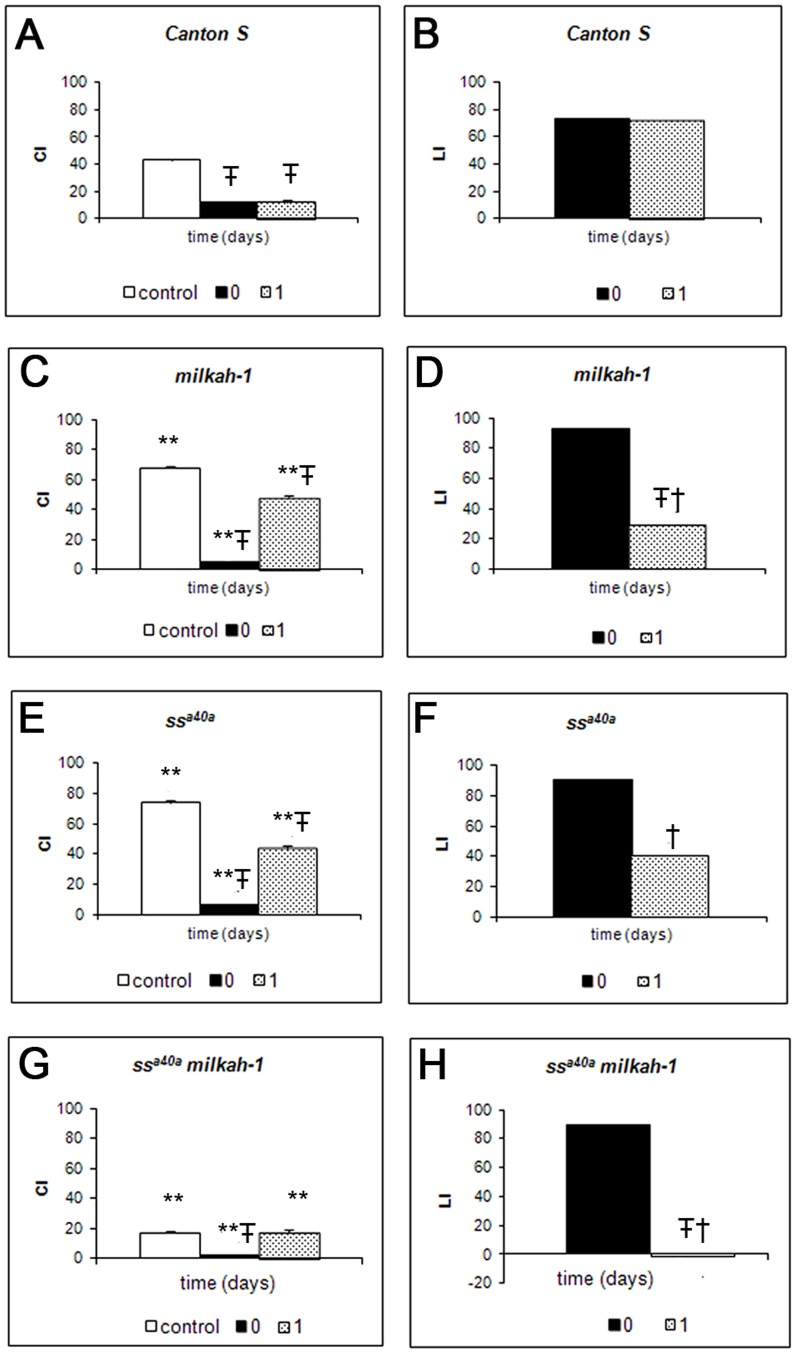
Dynamics of acquisition and retention of conditioned courtship suppression in mutant males. Males from wild type *Canton S* (A-B), *milkah-1* (C–D), *ss^a40a^* (E–F) and *ss^a40a^milkah-1* (G–H) lines were tested. (A, C, E, G) – 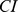 of tested males. (B, D, F, H) – 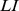
of tested males. On the X-axis: time following training, in days; on the Y axis: 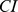 – courtship index, 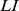
– learning index, standard units. Open columns – 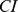 of naive males, hatched columns – 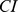 (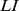) of trained males. Each point represents 20 males. ** - 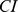 or 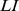
significantly lower than for wild type (two-sided randomization test, 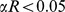) in similar condition; 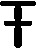 - 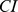 in a test immediately following training or in a deferred test, significantly lower than the CI of naive males (two-sided randomization test, 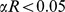); 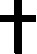 - 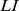
in the delayed test significantly lower than in test immediately following training (two-sided randomization test,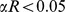.

We examined the effects of the *ss* and *CG5017* gene mutations using the conditioned courtship suppression paradigm (CCSP), which makes use of a natural conditioned reflex in insects – suppression of sexual activity as a result of accumulation of negative courtship experiences upon courting fertilized females. We compared the retention of CCS in *Drosophila* strains carrying hypomorphic mutations at the *spineless* and *CG5017* loci: *ss^a40a^*, *milkah-1* and their hybrids (Fig. 4). The 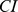
s of naive *ss^a40a^*, *milkah-1* and *ss^a40a^milkah-1* double mutants are lower than 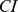
in *Canton S* wild type strain (two-sided randomization test, 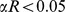). Although the mutant naive males of all three strains court well, are capable of immediate memory formation and of memory retention, the mutant males manifest defects in long-term memory retention, i.e. their CIs are restored one day after training (two-sided randomization test, 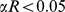). Since wild type males do not manifest such an increase in 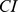 one day after training, this is indicative of long-term memory disruption in all three mutant strains. This effect is particularly strong in *ss^a40a^milkah-1* double mutant.

The dynamics of the Learning Index (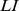) supports these conclusions. The 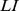
s of all three strains were significantly lower than that of wild type *Canton S* (two-sided randomization test, 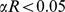). The LIs in all three mutant strains one day after training are significantly lower than in the test immediately after training (two-sided randomization test, 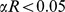). Since the 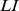
in wild type males does not decrease one-day after training, this is indicative of long-term memory disruption in mutant strains. Therefore, the dynamics of 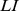
s is in concord with that of 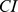
s, once more confirming the strong long-term memory defects especially in *ss^a40a^milkah-1* double mutant. Together, these results confirm the interaction between the *ss* and the *CG5017* genes revealed in behavioral paradigm.

## Discussion

Together, our results indicate that the *ss* and *CG5017* genes synergize to affect both morphogenesis and behavior in the fruit fly. Combination of a weak hypomorphic mutation in the *ss* gene with either of the two independently derived mutations in the *CG5017* (*milkah-1*) locus, results in profound changes in the structure of the leg and antennal structures of the fly. Moreover, combination of hypomorphic mutations in the *ss* and *CG5017* loci dramatically sensitizes the fly to the action of very low doses of ionizing radiation. This gene interaction is also revealed in a different experimental paradigm – courtship behavior of the fly and its ability to retain information.

Involvement of *CG5017 (milkah-1)* gene in learning and memory in the fly has been convincingly demonstrated using olfactory conditioning paradigm [16]. We here use a different behavioral paradigm (CCS) to demonstrate of *CG5017* in courtship behavior and memory. The advantage of this method is its natural and physiological nature, in contrast to method of learning through negative reinforcement (e.g., by electric shock or aversive odors). All three mutant lines retained the usual structure of courtship behavior, although hybrid *ss^a40a^milkah-1* flies had dramatically decreased 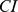 (Fig. 4). We also found that all three mutant lines showed high learning abilities. However, their ability to retain information 1 day later was disrupted. This disruption of memory was particularly evident in hybrid *ss^a40a^milkah-1* flies, where 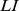 was close to zero (Fig. 4). Interestingly, we have also noticed an effect of the *ss* gene on memory in the CCS experiments; it will be interesting to know whether this effect is also revealed in other learning and memory paradigms. Together, these experiments provide additional evidence for the synergistic interactions between the ss and *CG507* genes.

While our results indicate genetic interactions, relations between the two genes may be also underpinned by direct molecular interactions. Ss protein is known to interact with Tango (Tgo), the fly homolog of mammalian Arnt and through this interaction proteins are getting transported to the nucleus and control gene expression by to the XRE motif in the promoter of defined set of genes [1]. The Ss::Tgo heterodimer can both suppress and activate specific genes, indicating heterodimer's interaction with other transcription or nucleosome assembly factors. With *CG5017* coding for a nucleotropic chaperone which belongs to a larger protein family that mediates nuclear transport and nucleosome assembly, its molecular interaction with the Ss or Tgo proteins, Ss::Tgo heterodimer, or other proteins that bind Ss, Tgo, or Ss::Tgo looks highly plausible.

We found that levels of *ss* gene transcription increase in response to furazidin and 5-hydroxytryptamine. It was previously shown that increase of *ss* gene ectopic expression led to abnormality of eye and leg morphogenesis [18]. Therefore one could expect that elevated levels of *ss* transcripts in response to furazidin and 5-hydroxytryptamine would disrupt eye and leg morphogenesis. Instead, we found that this increase of *ss* levels rescued the *ss^aSc^* leg phenotype but was accompanied by lethality (70% of larva and pupas died) induced by combined furazidin and radiation exposure.

Our results indicate that *ss* gene is involved in response to xenobiotics and radiation because mutated *ss*, particularly in combination with mutation in *CG5017*, significantly decreased the response of detoxifying genes *Cyp6g1 and CG1681*. *Drosophila ss* gene, in contrast to its homologues in higher vertebrates, lacks the capacity to bind to xenobiotic ligands such as TCDD [18], [30], [31]. This has cast doubt on its participation in the response to toxins. However, the changes in *Cyp*, *Gst* and *ss* gene transcription in response to furazidin and 5-hydroxytryptamine that we have demonstrated, suggest that the situation may be more complex. The basal level expression of the XRE-containing oxidative stress genes is lower in *ss* mutants compared to wild type flies (Fig. S1). Therefore, it is possible that *ss* mutants have lowered response to oxidative stress in general and are more susceptible to common oxidative stress reagents (xenobiotics). While we cannot confirm that these xenobiotics themselves serve as ligands that activate the Ss protein, it is conceivable that, as a result of their toxic action, an endogenous ligand capable of activating the Ss protein is generated. Such a role could be played by one of the endogenous AHR ligands – a toxic tryptophan derivative formyl-indolo-carbazole (FICZ). Cellular concentrations of FICZ increase significantly in response to ionizing radiation and this in turn stimulates increased expression of the *Ahr* gene [19].

It is possible that high level of mortality of larvae and pupae of the *ss^aSc^* line following combined action of furazidin and radiation is due to synergism of their toxic effects. The *ss^aSc^* line differs from the *ss^a40a^* line by the presence of a mutation in the *CG5017* gene, in addition to the mutation in the *ss* gene. Inasmuch as mutations in the *CG5017* gene do not disrupt morphogenesis, but do enhance the phenotypic manifestation of *ss* gene mutations and alter the levels of transcription of the *Cyp6g1, CG1681* and *ss* genes in response to the action of toxins, it is conceivable that its expression product is necessary for the complete functioning of the *ss* gene products. Since the *CG5017* gene codes for a nucleotropic chaperone [16], [17], this possibility looks plausible (although the molecular details of the *ss-CG5017* interaction remain to be elucidated).

Overall, our results suggest that *ss* and *CG5017* genes interact and together control fly morphogenesis, formation of long-term memory and cellular response to xenobiotic and radiation. Such interactions may have developed during the evolution of multi-cellular organisms in order to avoid toxicity in the environment and became employed by cells and organisms at various stages of development and vital activity. AHR-mediated response to potentially toxic components of environment may have facilitated both adaption to a new ecological niche and developing aversion to dangerous food ingredients at different steps of evolution [32], [33].

Together, our results show synergistic interactions between the *ss* and *CG5017* genes in *Drosophila*. These results may have broader implications beyond the model organism. In particular, they may indicate an increased risk of pathological response to radiation in humans carrying hypomorphic mutations of these genes in their genome (note that both genes are highly evolutionary conserved). Such individuals may be more vulnerable than the bulk of the population to even low levels of radiation, such as those delivered during routine medical procedures, prolonged air travel, or long term residency on the premises with high levels of radon. It is also plausible that such individuals may have increased sensitivity to pharmacological modifiers of the effects of radioactive exposure, which are often used in parallel with radiation. With the advent of broadly available genome sequencing it may become possible to identify such individuals and use additional measures to ameliorate their increased vulnerability to radiation.

## Supporting Information

Figure S1 **The basal level expression of** ***Cyp6G1*** **,** ***GST-theta*** **and** ***ss*** **genes in wild type and** ***ss^a40a^*** **and** ***ss^aSc^*** **mutant flies.** The relative level of expression of mRNA was measured using real-time PCR. The bars show the level of mRNA expression. The error bars represent the standard error of the mean of triplicate experiments.* - P<0.05, compared to *Canton S* group.
(TIF)

Table S1 **Quantification of leg phenotypes presented in** **Fig. 2** **.** Developmental conditions: Standard – larvae grown on the standard medium. Furazidin and 5-HT - larvae grown on the standard medium with 80 ml/L of furazidin or 5-hydroxytryptamine, respectively. 1-10 R and 500 R – larvae irradiated with a dose of 1-10 R or 500 R, respectively. 1-10 R + Furazidin and 1-10 R + 5-HT – larvae grown on the standard food with furazidin or 5-hydroxytryptamine and irradiated with a dose of 1-10 R (see Material and Methods). Legs were classified according to the number of tarsi present (from one to five). In each experiment 100 legs from at least 20 flies were observed per sample.
(DOCX)
