# Supplementary figures and images for: Combination of Hypomorphic Mutations of the Drosophila Homologues of Aryl Hydrocarbon Receptor and Nucleosome Assembly Protein Family Genes Disrupts Morphogenesis, Memory and Detoxification
Source: PLoS One. 2014 Apr 15;9(4):e94975. doi: 10.1371/journal.pone.0094975 (PMC3988104; doi:10.1371/journal.pone.0094975)

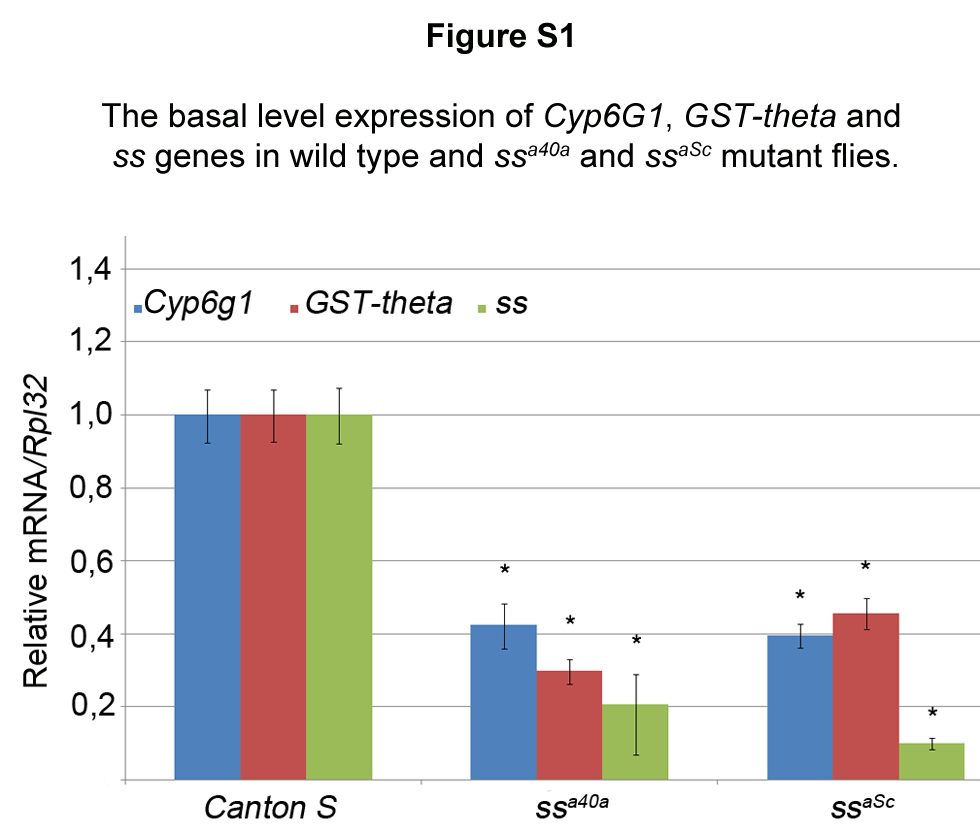

Supplement: Figure S1 — The basal level expression of Cyp6G1 , GST-theta and ss genes in wild type and ssa40a and ssaSc mutant flies. The relative level of expression of mRNA was measured using real-time PCR. The bars show the level of mRNA expression. The error bars represent the standard error of the mean of triplicate experiments.* - P<0.05, compared to Canton S group. (TIF) [file pone.0094975.s001.tif]
