# Supplementary material for: Combination of Hypomorphic Mutations of the Drosophila Homologues of Aryl Hydrocarbon Receptor and Nucleosome Assembly Protein Family Genes Disrupts Morphogenesis, Memory and Detoxification
Source: PLoS One. 2014 Apr 15;9(4):e94975. doi: 10.1371/journal.pone.0094975 (PMC3988104; doi:10.1371/journal.pone.0094975)
Supplement: Table S1 — Quantification of leg phenotypes presented in Fig. 2 . Developmental conditions: Standard – larvae grown on the standard medium. Furazidin and 5-HT - larvae grown on the standard medium with 80 ml/L of furazidin or 5-hydroxytryptamine, respectively. 1-10 R and 500 R – larvae irradiated with a dose of 1-10 R or 500 R, respectively. 1-10 R + Furazidin and 1-10 R + 5-HT – larvae grown on the standard food with furazidin or 5-hydroxytryptamine and irradiated with a dose of 1-10 R (see Material and Methods). Legs were classified according to the number of tarsi present (from one to five). In each experiment 100 legs from at least 20 flies were observed per sample. (DOCX) [file pone.0094975.s002.docx]

**[Table S1](http://www.plosone.org/article/info%3Adoi%2F10.1371%2Fjournal.pone.0015382#pone.0015382.s001). Quantification of leg phenotypes presented in Fig. 2.**

| **Developmental** | **n. legs with a given number of tarsi** | | | | | **Lethality** |
| --- | --- | --- | --- | --- | --- | --- |
| **condition** | **one** | **two** | **three** | **four** | **five** | **(%)** |
| **Genotype: wild type or *ss^a40a^* or *CG5017*** | | | | | | |
| Standard | **-** | **-** | **-** | **-** | 100 | 0 |
| Furazidin | **-** | **-** | **-** | **-** | 100 | 0 |
| 5-HT | **-** | **-** | **-** | **-** | 100 | 0 |
| 1-10 R | **-** | **-** | **-** | **-** | 100 | 0 |
| 500 R | **-** | **-** | **-** | **-** | 100 | 0 |
| 1-10 R + Furazidin | **-** | **-** | **-** | **-** | 100 | 0 |
| 1-10 R + 5-HT | **-** | **-** | **-** | **-** | 100 | 0 |
| **Genotype: *ss^aSc^* (*ss^a40a^CG5017*)** | | | | | | |
| Standard | **-** | **-** | 18 | 82 | - | 0 |
| Furazidin | **-** | **-** | - | - | 100 | 0 |
| 5-HT | **-** | **-** | - | - | 100 | 0 |
| 1-10 R | 62 | 29 | 9 | - | - | 0 |
| 500 R | 80 | 15 | 5 | - | - | 0 |
| 1-10 R + Furazidin | **-** | 4 | 16 | 80 | - | 70 |
| 1-10 R + 5-HT | **-** | **-** | - | 81 | 19 | 0 |
